# The Quest for Cellular Prion Protein Functions in the Aged and Neurodegenerating Brain

**DOI:** 10.3390/cells9030591

**Published:** 2020-03-02

**Authors:** Rosalina Gavín, Laia Lidón, Isidre Ferrer, José Antonio del Río

**Affiliations:** 1 Molecular and Cellular Neurobiotechnology, Institute for Bioengineering of Catalonia (IBEC), Barcelona Institute of Science and Technology (BIST), Science Park of Barcelona, 08028 Barcelona, Spain; llidon@ibecbarcelona.eu (L.L.); jadelrio@ibecbarcelona.eu (J.A.d.R.); 2 Department of Cell Biology, Physiology and Immunology, Faculty of Biology, University of Barcelona, 08028 Barcelona, Spain; 3 Center for Networked Biomedical Research on Neurodegenerative Diseases (Ciberned), 28031 Barcelona, Spain; isidreferrer@ub.edu; 4 Institute of Neuroscience, University of Barcelona, 08028 Barcelona, Spain; 5 Department of Pathology and Experimental Therapeutics, University of Barcelona, 08907 Barcelona, Spain; 6 Senior Consultant, Bellvitge University Hospital, Hospitalet de Llobregat, 08907 Barcelona, Spain; 7 Bellvitge Biomedical Research Institute (IDIBELL), Hospitalet de Llobregat, 08908 Barcelona, Spain

**Keywords:** prion, Tau, Alzheimer’s disease, Parkinson’s disease, Huntington’s disease, neuroprotection

## Abstract

Cellular (also termed ‘natural’) prion protein has been extensively studied for many years for its pathogenic role in prionopathies after misfolding. However, neuroprotective properties of the protein have been demonstrated under various scenarios. In this line, the involvement of the cellular prion protein in neurodegenerative diseases other than prionopathies continues to be widely debated by the scientific community. In fact, studies on knock-out mice show a vast range of physiological functions for the protein that can be supported by its ability as a cell surface scaffold protein. In this review, we first summarize the most commonly described roles of cellular prion protein in neuroprotection, including antioxidant and antiapoptotic activities and modulation of glutamate receptors. Second, in light of recently described interaction between cellular prion protein and some amyloid misfolded proteins, we will also discuss the molecular mechanisms potentially involved in protection against neurodegeneration in pathologies such as Alzheimer’s, Parkinson’s, and Huntington’s diseases.

## 1. Introduction

Pathogenic conformational changes of cellular prion protein (PrP^C^) generates a β-sheet-enriched isoform called PrP^Sc^ or prion (word derived from proteinaceous infectious particle) [1,2,3], the causal agent of prionopathies [1,2]. PrP^C^, the natural noninfective protein, is a cell surface glycoprotein linked to the membrane by a glycosylphosphatidylinositol (GPI) anchor, and is mainly located in lipid rafts. The protein is encoded by the *Prnp* gene and expressed in a wide range of tissues in mammals [4,5,6]. However, central nervous system (CNS) and lymphoid tissues express higher levels of PrP^C^, making them the best candidates to generate infectious prions. Prionopathies are a group of fatal neurodegenerative diseases (NDDs) that may present as genetic, infectious, or sporadic disorders. Kuru, Creutzfeldt–Jackob disease (CJD), Gertsmann–Straussler–Scheinker syndrome (GSS), and fatal familial insomnia (FFI) are diseases that occur in humans, while bovine spongiform encephalopathy (BSE) is found in cows, scrapie in sheep, and chronic wasting disease (CWD) in some members of the family Cervidae [7,8,9]. After the structural transition of PrP^C^, PrP^Sc^ acquires self-aggregative, spreading (intercellular propagation), and infective (understood as a synonym of contagious) properties [10,11].

In recent years, the scientific community has focused attention on defining the term “prion-like” or “prionoid” to describe other proteins with behavior similar to prions in terms of self-aggregation and spreading properties [10,11,12,13,14]. Proteins implicated in different NDDs, including Huntingtin [15], α-synuclein [16,17,18,19,20,21,22,23], amyloid-β (Aβ) [24,25,26], and tau [27,28,29,30,31], are currently seen in numerous studies as prion-like proteins. In fact, most of them display amino acid domains in their sequence that determine their specific self-aggregation properties [32,33]. However, the transmission of some of these amyloid proteins between individuals, although unlikely, is currently a relevant topic for discussion [34,35,36].

**Figure 1:**
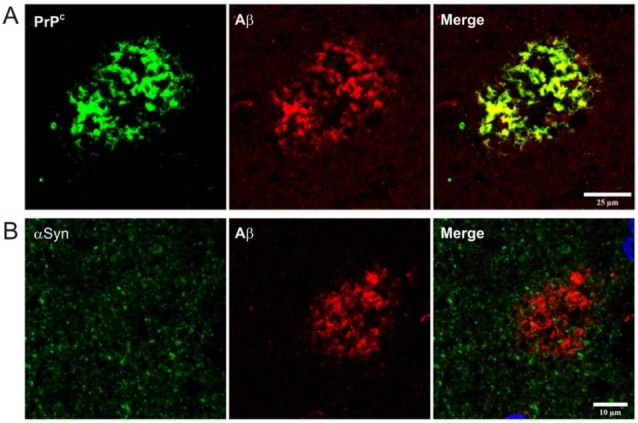
Confocal images of self-aggregative proteins in AD patients. (**A**) Double-labeling immunofluorescence of PrP^C^ (clone 3F4 directed against aa 109–112 of prion protein, Merck Millipore) and Aβ (rabbit polyclonal antibody directed against the N-terminus 11-pyro E start point of human beta-amyloid, Novus Biologicals) showing colocalization of PrP^C^ in Aβ deposits. (**B**) Double-labeling immunofluorescence of α-synuclein (clone 5C2 raised against recombinant alpha-synuclein aa 61–95 purified from *E. coli*, Labome) and Aβ (Novus). Note the absence of clear colocalization between these two proteins. Scale bar values are displayed in the Merge panels.

More relevantly, two or more of these proteinaceous species might coexist in particular NDDs or experimental models (i.e., [37,38,39,40,41,42,43,44,45]). Thus, the molecular interaction between them and their putative synergistic effects in affected patients are still under debate (i.e, tau and α-synuclein) [46]. However, the question arises when we try to ascertain natural PrP^C^ functions and their specific roles in NDDs other than prionopathies because not only PrP^Sc^ (see above) but also PrP^C^ can coexist with most of these amyloids in experimental NDD models and in brain affected tissue (i.e., [22,47,48,49,50]). In this sense, Figure 1A illustrates an example of the relevant colocalization between PrP^C^ (green) and Aβ (red) in the postmortem frontal cortex of an Alzheimer’s disease (AD) patient in contrast to other prion-like proteins such as α-synuclein with little colocalization with Aβ-positive plaques in the same context; normal endogenous expression and function of PrP^C^ may be largely compromised in NDDs [51,52]. In this respect, conflicting studies report neurotoxic roles of PrP^C^ in particular NDDs while others point to a neuroprotective function of the protein in the same disease (i.e., AD), discussed below.

In the present review, far from arguing the pathogenicity of PrP^Sc^ or other prion-like proteins, which has already been done in several reviews (see, for instance, [53,54,55,56]), we will focus our attention on the neuroprotective role of PrP^C^ and its putative implication in amyloid-associated NDDs.

## 2. PrP^C^ and Neuroprotection

Since the generation of the first knock-out (*Prnp^0^*^/*0*^) mice of PrP^C^ in 1992, referred to as Zürich 1 [57], researchers have used different *Prnp^0^*^/*0*^ mice in their studies (Zürich 1 [58], Edinburgh [59], and Zürich 3 [60]) to validate/reveal processes sustained by the functions of the protein (see also [61,62]). Moreover, the number of functions is paralleled by the numerous descriptions of PrP^C^ interactions [63,64,65,66]. However, some attributed functions are controversial to interpret in biological terms [23,63,67,68]. We refer the reader to a compelling description of PrP^C^ interactions [60,69,70] in order to evaluate the pitfalls associated with the genetic background of the Zürich 1 mice. However, one of the most frequently described functions in different experimental models is the participation of PrP^C^ in neuroprotection. In these processes, the “positive” actions of the protein are linked to: i) particular PrP^C^ activities and ii) PrP^C^ interaction with counterpart actors modulating cell signaling cascades and mechanisms involved in neurotoxicity.

### 2.1. Antioxidant Activity

Membrane-anchored PrP^C^ presents the ability to bind extracellular Cu^2+^ ions at the highly conserved octapeptide repeats (OR) region of the protein near the N-terminus [71,72,73,74]. This PrP^C^–Cu^2+^ interaction provides antioxidant properties to the protein as demonstrated by reducing copper-mediated oxidative stress [75]. In fact, *Prnp^0^*^/*0*^ mice display higher levels of oxidative stress markers in vivo compared to wild-type animals [76,77,78]. Moreover, in vitro studies have shown that PrP^C^ overexpression in cultured non-neuronal cells results in decreased susceptibility to oxidative damage and toxicity induced by agents such as copper and hydrogen peroxide (H_2_O_2_) [79,80,81,82,83,84]. In fact, PrP^C^ levels are increased in neuroblastoma (N2a) and HeLa cells after overload of extracellular copper [85]. Stress-mediated overexpression of PrP^C^ might modulate superoxide dismutase (SOD) [86] and glutathione reductase (GR) activity [83], increasing antioxidant properties in treated cells.

The antioxidant role of PrP^C^ has also been reported in vivo. In this regard, following ischemic insults, *Prnp^0^*^/*0*^ mice display larger affected regions with increased cell death than do wild-type mice [87]. In contrast, endogenous PrP^C^ expression can protect against brain damage after traumatic brain injury in mice [88] and during stroke in rats [89,90,91]. In parallel, as a consequence of the lesion, *Prnp* expression is increased under oxidative stress conditions [92], and both increased mRNA and protein have been described in neurons located in the penumbra region in ischemic mice [87]. Lastly, the above-mentioned regulation has also been described in humans under oxidative damage or ischemia [87,93]. Overexpression of PrP^C^ by affected brains should be considered an intrinsic response in an attempt to provide long-term neuroprotection, neurogenesis, or angiogenesis [94].

### 2.2. Antiapoptotic Activity

PrP^C^ not only acts as an antioxidant protein but also exercises direct control on mitochondrial-associated apoptotic signaling. In this sense, overexpression of PrP^C^ protects primary cultured neurons against Bax-mediated cell death [95]. Function-mapping studies have reported that the OR domain of PrP^C^ is mandatory for antiapoptotic function, since deletion of this domain abolishes the protective function of the protein. Other antiapoptotic proteins such as Bcl-2 require the BH2 domain to interact with Bax protein and regulated permeabilization of the mitochondrial membrane [96]. Although PrP^C^ does not contain BH2 domains and does not directly interact with Bax, it is able to bind with the C-terminal domain of Bcl-2 [97,98]. Moreover, ectopic expression of PrP^C^ and Bcl-2 in *Prnp*^0/0^ cells suppresses apoptosis in serum-free conditions [80,99], suggesting the contribution of PrP^C^ to antiapoptotic activity through caspase-dependent apoptotic pathways in mitochondria.

### 2.3. Regulation of Calcium Homeostasis and Ionotropic Glutamate Receptors by PrP^C^

Alterations in calcium metabolism have been extensively studied in acute injuries such as ischemia and in neurodegeneration. Relevantly, PrP^C^ may regulate intracellular Ca^2+^ homeostasis [100,101,102]. For example, Krebs et al. showed that endoplasmic reticulum (ER) calcium stores respond to H_2_O_2_ in a PrP^C^-dependent way in neuronal cell cultures [103]. The data presented support the relevance of the OR domain of the protein in this function and the activation of a protective signaling cascade involving Src-like tyrosine kinase Fyn. Activated Fyn may further activate cellular phospholipases to generate inositol triphosphate (IP3), leading to the opening of ER-associated calcium channels [104].

In fact, a neuroprotective function of PrP^C^ is also supported by several studies describing increased neuronal susceptibility to glutamate agonists in *Prnp^0^*^/*0*^ mice (see, for instance, [105,106,107]). In these studies, AMPA/KA receptor dysfunction, largely responsible for excitotoxicity, is highlighted by the absence of PrP^C^. Indeed, recent studies have also reported that PrP^C^ binds to and modulates N-methyl-D-aspartate receptor (NMDAR) in cooperation with Cu^2+^ [108,109,110]. In addition, there is a susceptibility to stroke in rats with downregulation of *Prnp* and an increased expression of NR2B, a subunit of NMDAR implicated in excitotoxicity-induced neuronal apoptosis [111]. This suggests a functional regulation of ionotropic glutamate receptors by PrP^C^. The reader can find additional information about the mechanisms implicated in neuroprotection of excitotoxicity by PrP^C^ in recent reviews [112,113].

### 2.4. Molecular Partners of PrP^C^ for Interaction and Cell Signaling

The ability of PrP^C^ to bind a variety of other molecules suggests the existence of different physiological roles, which may be context-specific. In this respect, PrP^C^ could be considered a cell surface scaffold protein [70] as a means to explain the role of the protein as key in different signaling systems. However, it will be necessary to determine the biological significance of each interaction and the possibilities of response depending on these contacts. In terms of neuroprotection, PrP^C^ transduces signals across the plasma membrane by binding to other plasma membrane molecules such as the laminin receptor and neural cell adhesion molecule. These interactions promote cell survival and neurite outgrowth [114,115]. In this sense, cell survival promoted by PrP^C^ can involve the activation of both cyclic adenosine monophosphate/protein kinase A (cAMP/PKA) and the ERK1/2 signaling pathways [116], as well as activation of the phosphatidyl-inositol-3-kinase/Akt pathway [115]. In addition, Moulliet-Richard and colleagues proposed Fyn kinase as an initiator of the cascade leading to ERK1/2 activation by cross-linking of PrP^C^ at the cell membrane with antibodies [117]. These and other results support the idea that dimer formation of PrP^C^ is essential to its neuroprotective activity since antibody-mediated PrP^C^ dimerization elicits rapid phosphorylation of ERK1/2 in cultured cells [118]. Moreover, in the same in vitro model, PrP^C^ dimerization also promotes the recruitment of the cAMP responsive element-binding protein (CREB) transcription factor and the transcription of several genes with key functions in cellular protection and neuronal plasticity [119]. In addition, GSK3β, whose inhibition is neuroprotective, is a downstream target of PrP^C^ dimerization signaling in serotonergic neuronal cells [120]. However, dimer formation of PrP^C^ seems to be necessary but not sufficient for its stress protective activity [121].

Another neuroprotective pathway through PrP^C^ binding with stress-inducible protein 1 (STI-1) has been reported by [122]. Moreover, Lopes and colleagues showed the effects on both neuritogenesis and survival in hippocampal neurons triggered by STI-1-PrP^C^ interaction. In this regard, the neuritogenesis was found to be dependent only on mitogen-activated protein kinase (MAPK) activity, whereas cAMP-dependent PKA actions mediate neuroprotection [123]. In addition, PrP^C^ cooperates with STI-1 to regulate SOD activity, and consequently cell survival [124]. In fact, a recent study demonstrates the neuritogenic potential of recombinant PrP^C^ [125] which might trigger intracellular signaling cascades after its homophilic interaction with membrane-anchored PrP^C^. Indeed, intracellular endocytosed PrP^C^ may interact with proteins involved in classical signaling pathways including the growth factor receptor-bound protein 2 (Grb2), an adaptor protein involved in neuronal survival [63,126].

### 2.5. Physiological Processing of PrP^C^ and Neuroprotective Metabolites

**Figure 2:**
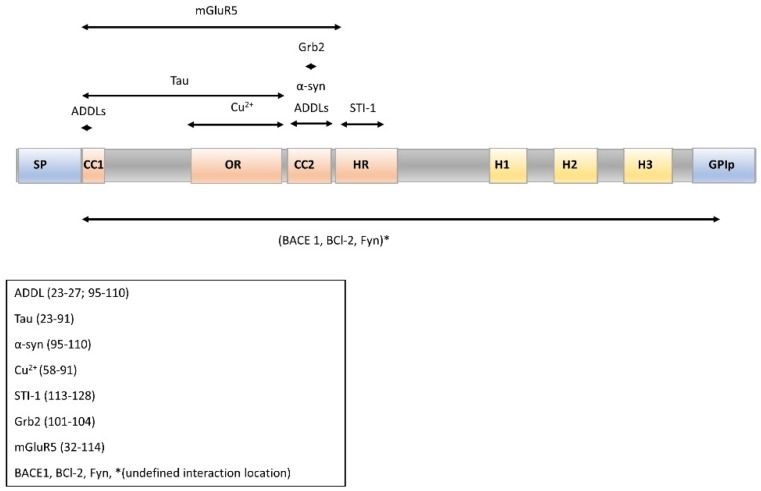
Schema of the PrP^C^ sequence with its different domains showing the interaction of molecules described in this article. SP: signal peptide 1–22 aa; CC1: charged cluster 1,23–30 aa; OR: octarepeat region 60–91 aa; CC2: charged cluster 2, 95–110 aa; HR: hydrophobic region 112–133 aa; H1-3: α-Helix regions 143–452, 171–191, 199–221; GPIp: GPI anchor-signaling peptide. (Numbering based on the moPrP sequence.)

**Figure 3:**
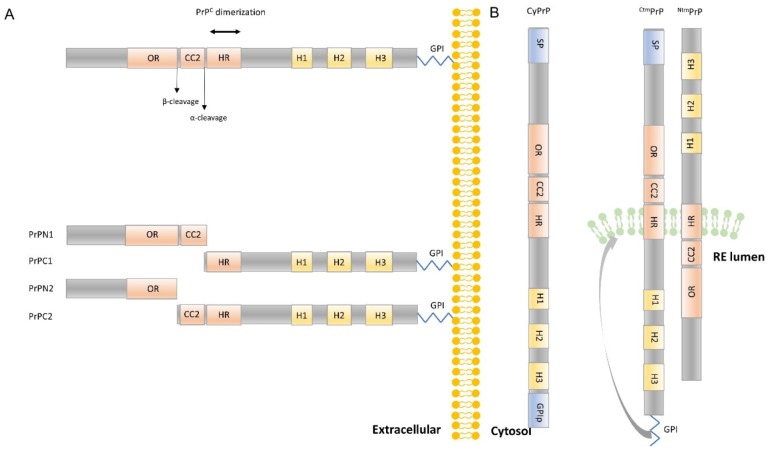
Schematic representation of PrP^C^ isoforms: (**A**) Cell surface PrP^C^ and the four metabolites resulting from α- and β-cleavage (PrPN1, PrPC1, PrPN2, PrPC2, respectively). Homodimerization of PrP^C^ enhanced α-cleavage and consequently the production of anti-β activity of PrPN1. (**B**) Cytosolic PrP (CyPrP) and the two transmembrane isoforms termed ^Ntm^PrP and ^Ctm^PrP with opposite sequence orientations with respect to the lumen of the endoplasmic reticulum. As the CC1 domain is not involved in the generation of different PrP^C^ isoforms, it has not been included in this figure.

PrP^C^ can be proteolytically cleaved in the two structurally different regions of the protein at the plasmatic membrane. PrP^C^ has a long, flexible N-terminal tail (residues 23–128) and a C-terminal globular domain that contains three α-helices and two parallel stranded β-sheets [127,128,129] (Figure 2) (the residue numbering refers to mouse PrP (moPrP)). These two regions follow physiological proteolytic processing by α-cleavage (approximately at aa 110), releasing the PrPN1 fragment (aa 23–110) and yielding the PrPC1 fragment tethered to the plasma membrane [130,131]. In addition, minor cleavage, termed β-cleavage, occurs at residues 90–91, releasing aa 23–89 or PrPN2 and PrPC2 [131] (Figure 3A). A third type of cleavage within the OR is induced by reactive oxygen species in the presence of Cu^2+^ [132]. One study [133] showed neuroprotective activity and anti-β-sheet-mediated corruption activity of PrPN1. In this sense, different studies reinforce this hypothesis [134,135,136], and neurotoxic consequences of the absence of α- or β-cleavage-derived forms of PrP^C^ have been reported [137,138]. In fact, PrP^C^ homodimerization stimulates its trafficking to the plasma membrane and α-cleavage with the consequent production of PrPN1 and PrPC1 [136].

In addition, a great range of ligands binding to the N-terminal domain of PrP^C^ are able to trigger rapid endocytosis of the protein. Ligand-induced internalization of PrP^C^ may protect cells in different ways: through the transport and homeostasis of several ligands, including Cu^2+^ and hemin [75,139], by degradation of misfolded or inactive PrP^C^ molecules [140], and by activating PrP^C^-mediated intracellular cell signaling after the stimuli [141,142,143]. In this regard, and under physiological conditions, endocytosis is essential to N-terminal PrP^C^ function, although PrPN1 is able to elicit neuroprotective signals independently of internalization [134].

Alternatively, intracellular processing of PrP^C^ could generate additional cytoplasmic forms. A cytosolic form (CyPrP) can be generated, probably as a result of retrotranslocation from the ER or from poor translocation into the ER [144]. CyPrP is proposed as being responsible for protection against Bax-mediated cell death [145,146]. In addition, PrP^C^ presents two transmembrane isoforms, termed ^Ntm^PrP and ^Ctm^PrP, with opposite sequence orientations with respect to the lumen of the ER [147]. The single-pass transmembrane isoforms represent 2% of total PrP^C^ inserts [148] (Figure 3B), and to date, no physiological function has been detailed for them. However, several studies have associated overexpression of ^Ctm^PrP with neurotoxicity [149,150].

## 3. Functions of PrP^C^ during Aging and Neurodegeneration

In the healthy brain, there is a relationship between PrP^C^ levels and aging. In fact, protein levels are reduced in older human brains [151]. In this sense, aging is associated with an increase in reactive oxygen species (ROS) which inversely correlates with PrP^C^ levels. Moreover, oxidative stress is an important contributing factor in the pathogenesis of many human NDDs, such as prionopathies, Parkinson’s, Huntington’s, Alzheimer’s, and amyotrophic lateral sclerosis [152,153,154], which leads us to pose the question of what the role of PrP^C^ is in each of these scenarios. In addition, as mentioned above, there is a specific interaction between the principal proteins implicated in these diseases and PrP^C^, for instance, tau [46,48], Aβ [155,156], and α-synuclein [22,23,157], reinforcing the notion of an active role for PrP^C^ in these pathologies. Table 1 summarizes several studies describing examples of the neuroprotective role of PrP^C^ in a number of neurodegenerative diseases and some nonspecific disorders. Please see text for opposing data.

### 3.1. PrP^C^ in Alzheimer’s Disease (AD) and Other Tauopathies

AD patients are characterized by a progressive cognitive decline and behavioral changes due to the dysregulation of two prion-like proteins: (i) Aβ, resulting from the abnormally processed amyloid precursor protein (APP) though greater activity of β-secretase-1 (BACE1), and (ii) tau, a microtubule-associated protein that promotes the polymerization and stabilization of microtubules (MT) under the regulatory control of several kinases and phosphatases [171,172]. As a result, the major histopathological hallmarks of the disease are the presence of senile plaques, enriched in Aβ, and neurofibrillary tangles (NFTs) containing hyperphosphorylated tau (e.g., [173,174]). The widely accepted amyloid cascade hypothesis posits that the generation of Aβ and its extracellular deposition in brain parenchyma triggers a sequence of events leading to tau dysfunction following the “staging” theory of disease progression [175,176]. Although higher toxic potential is actually attributed to Aβ-derived diffusible ligands (ADDLs) and not to insoluble forms of Aβ [177], tau is considered decisive for the progression of neurodegeneration [178], and the spreading of the tau pathology in affected individuals correlates well with memory impairment and dementia symptoms [179]. Other tauopathies, with different target cells (from neurons to astroglia or oligodendroglia) include Pick’s disease (PiD), progressive supranuclear palsy (PSP), corticobasal degeneration (CBD), and argyrophilic grain disease (AGD), among others [180,181]. See also [182,183] for recent classifications of tauopathies.

The tau gene (*MAPT*) is expressed in six isoforms as a result of mRNA alternative splicing in various combinations, distinguishable by the exclusion or inclusion of a repeat region of exon 10 that generates four microtubule-binding repeats (4R) or three (3R) tau, and both with either no (0N), one (1N), or two (2N) amino-terminal inserts [184]. In adult neurons, 3R and 4R tau isoforms are present to a similar degree, localized mainly in axons, but they are also present in the somatodendritic compartment of neurons underlying NFT formation and in AD. NFTs lead to increased MT instability, impaired axonal transport, and profound deficits in synaptic function. Among others, GSK3β and Cdk5 are the main kinases implicated in the phosphorylation of some tau epitopes described in AD [185,186,187,188]. In fact, several of these phosphorylated tau epitopes have been associated with in vitro cellular response to ADDLs [167,189,190]. 

In AD, there is a colocalization of PrP^C^ and Aβ-containing senile plaques ([49] and Figure 1A). Moreover, PrP^C^ and ADDLs interact specifically in AD patients’ brains [50,191,192], suggesting an active role for PrP^C^ in the disease. In fact, Aβ oligomers influence PrP^C^ trafficking and inhibit PrP^C^ endocytosis [193], blocking BACE1 regulation by PrP^C^ [194,195]. In addition, a study by Strittmatter’s laboratory pointed to ADDL–PrP^C^ interaction as being responsible for neurodegeneration through regulation of glutamate receptors [50]. Later, other studies reinforced this hypothesis [196,197,198,199]. In the effort to explain the consequences of PrP^C^–ADDLs in glutamate receptor interaction, some groups have shown a physical connection between PrP^C^ and ionotropic glutamate receptor NMDA [197,200] and metabotropic glutamate receptor 5 (mGluR5) [201] (reviewed in [202]). NMDA receptor activity is modulated by PrP^C^ in a copper-dependent manner. Moreover, Um and colleagues linked the dysregulation of NMDA by ADDLs–PrP^C^ with Fyn activation. In fact, Fyn has also been associated with PrP^C^ [203] which presents direct binding to mGluR5, mGluR1, and NMDA receptors as well as tau [197,204,205,206,207], supporting a role for it in dysregulating NMDA- or mGluR5-mediated synaptic function as well as tau hyperphosphorylation induced by Aβ [208]. This was demonstrated by Lesne’s laboratory who showed that soluble Aβ binds to PrP^C^ at neuronal dendritic spines where it forms a complex with Fyn and causes tau hyperphosphorylation [209], which may lead to cell death. Regarding synaptic changes and neuronal physiology, some studies reported different data from those reported by Strittmatter´s lab. Indeed, some authors have observed Aβ-induced depression of synaptic transmission in both wild-type and *Prnp^0^*^/*0*^ mouse slices [210], and others have found that *Prnp^+^*^/*+*^ and *Prnp^0^*^/*0*^ mice were equally susceptible to cognitive impairment after Aβ injection into the lateral ventricle [211]. Moreover, Aguzzi’s group has shown there to be no LTP impairment in APP/PS1 mice lacking *Prnp* [212]. In summary, although the binding of PrP^C^ and ADDLs seems to be accepted, there are some differences in ascertaining whether this interaction affects only synaptic plasticity or cell death as well.

Below we describe several studies in which PrP^C^ developed a neuroprotective role in AD through its natural function/s. For instance, it has been reported that overexpression of PrP^C^ protects against Aβ-mediated cell death (i.e., caspase-3 activation) in mice via control of the Bax/Bcl-2 ratio and, over time, PrP^C^ expression also prevents cognitive dysfunction [165]. In addition, Younan and colleagues have shown that PrP^C^ inhibits fiber formation by trapping free ADDLs and causing disassembly of preformed Aβ fibrils [213]. The authors point to two charged clusters in the N-terminal domain of PrP^C^ as being responsible for Aβ–PrP^C^ binding: (aa 95–110) and (aa 23–27) [21,155,213]. At this point, it is important to keep in mind that the activity of PrP^C^ is finely regulated by its dimerization and that PrP^C^ homodimers stimulate the production of PrPN1, which in turn can bind to Aβ with high affinity, blocking transformation into soluble and toxic ADDLs [136,160,161,164]. In this sense, there is some evidence that α-cleavage (leading to PrPN1 and PrPC1) is increased in postmortem brains of AD patients, reinforcing this neuroprotective notion [160], and inhibition of PrPN1 production promotes AD progression [214]. The hydrophobic domain of the protein (amino acids 112–133), inside the N-terminal domain, is responsible for homodimer formation [121] and perhaps interferes with ADDL binding. In addition, although PrP^C^ could mediate the toxicity of ADDLs [133,215], homodimerization and cleavage may be a common mechanism in preventing this.

In addition, some authors have recently shown that increased PrP^C^ expression downregulates tau protein [167,216,217]. In this sense, we recently reported increased susceptibility of tau phosphorylation to ADDLs in primary cortical cultures lacking PrP^C^. Reported results indicate that increased PrP^C^ between Braak I and IV stages renders lower tau and phospho-tau. In contrast, PrP^C^ levels decreased at Braak V–VI stages which also correlates with increased amounts of tau and phospho-tau. Taken together, our observations suggest a protective role for PrP^C^ in early stages of AD which may be extendable to other tauopathies [167]. In fact, tau pathology has been reported in a wide number of prionopathies such as sporadic CJD (sCJD) [35], GSS [218,219], and FFI [220], which showed lower PrP^C^ levels due to the PrP^C^-to-pathogenic-prion conversion [221]. 

Regarding pathological phosphorylation of tau, some studies point to GSK3β kinase activity as a key element in neuroprotection, while GSK3β inhibition has been shown to play a pivotal role in synaptic plasticity and long-term potentiation (LTP) [222]. In this sense, PrP^C^–STI-1 interaction triggers reduction of GSK3β kinase activity which not only may affect tau phosphorylation but may also induce memory impairment [120]. Importantly, the interaction of STI-1 with PrP^C^ was recently shown to hinder the binding of Aβ oligomers to PrP^C^, overcoming their toxicity [162].

**Figure 4:**
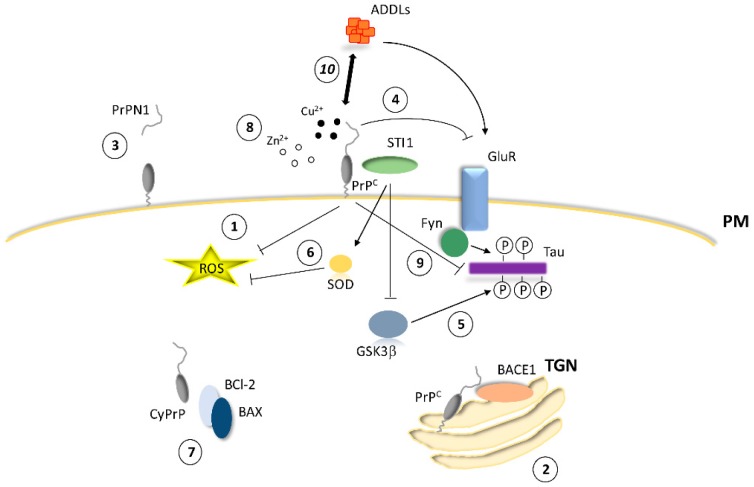
Proposal of a putative scenario for neuroprotective intervention of PrP^C^ in AD: **1**. Modulating ROS levels; **2**. Inhibiting BACE1 activity; **3**. Generating PrPN1; **4**. Modulating glutamate receptors (both ionotropic (NMDAR) and metabotropic (mGluR5)); **5.** Reducing phospho-tau levels through STI-1 interaction and GSK3β inhibition; **6**. Reducing ROS levels through STI-1 interaction and consequent SOD modulation; **7.** Executing anti-Bax activity; **8**. Increasing Zn^2+^ uptake; and **9.** Reducing tau levels. Number **10**, in italics, represents the direct intervention of ADDLs in PrP^C^ function, inhibiting its endocytosis and/or homodimerization, and competing with Cu^2+^ binding and homeostasis. TGN: Trans-Golgi Network.

As indicated by several studies, AD (in a broad sense) is characterized by neuroinflammation and oxidative stress [223]. In this sense, it is notable that levels of PrP^C^ are increased between Braak I and IV stages [167], in contrast to decreased levels in advanced stages of the disease [221,224]. PrP^C^ can reduce ROS by its intrinsic copper buffering roles and by modulating SOD1 and GR (see above). Elevated levels of PrP^C^ have been reported to occur in brain regions prone to oxidative stress in AD, suggesting a possible antioxidant function in the disease [166]. In addition, increased expression of the PrP^C^ in the first stages of AD [225,226] may promote competition between different ligands including Aβ (Figure 4). Along this line, zinc is another cation involved in the generation of ROS in neurons, and PrP^C^ mediates uptake of extracellular zinc into neuronal cells [227]. Furthermore, zinc promotes Aβ aggregation and increases insoluble Aβ and its deposition in plaques in an AD mouse model [228,229]. In addition, synaptic zinc favors the attachment of Aβ to NMDAR, mediating its excitotoxicity [230]. This implies that the reduction in PrP^C^ in the advanced AD brain migh result in decreased zinc uptake and, consequently, in an increase in the amount of zinc in the synaptic cleft, which would promote Aβ aggregation and synaptic targeting, thereby accelerating the neurodegenerative process.

PrP^C^ has been shown to influence the processing of APP, lowering Aβ production through inhibition of BACE1, suggesting that PrP^C^ functions are beneficial in AD [158,159]. In fact, protein and mRNA levels of PrP^C^ correlate inversely with BACE-1 activity and Aβ levels [151,158,221,226,231,232]. Therefore, a decrease in PrP^C^ levels at medium-late stages of AD may be a primary contributor to neurodegeneration and cognitive impairment.

### 3.2. Neuroprotective Role of PrPC in Huntington’s and Parkinson’s Diseases

Despite the paucity of data supporting this hypothesis, assorted evidence suggests that PrP^C^ is a possible neuroprotective key in other diseases, since ROS and free radicals are important mediators of neurotoxicity in several other NDDs—for instance, HD and PD (see above), to which we will now turn.

Huntington’s disease (HD) is an inherited disorder which causes progressive neurodegeneration and which includes motor, cognitive, and psychiatric manifestations until inevitable death occurs [233]. The disease is caused by a polyglutamine (polyQ) expansion (encoded by a CAG repeat) of Huntingtin (HTT) protein. Mutated HTT gene is responsible for the aggregated polyQ, the main component of the proteinaceous deposits found in patient brains [234]. In fact, the age of onset of clinical manifestations is inversely correlated to the length of the polyQ expansion. HTT is expressed in a broad spectrum of neuronal and non-neuronal tissues [235]. Nevertheless, mutated HTT protein promotes progressive neurodegeneration of specific neuronal types, affecting particularly the caudate-putamen and neocortical regions of HD patient brains [236,237]. However, the mechanism of progressive neural loss has not been fully elucidated [238].

Parkinson’s disease (PD) is the second most common neurodegenerative disorder in the world, and it is characterized by the appearance of postural instability, bradykinesia, and tremor. These symptoms are associated with dopaminergic neurodegeneration of the substantia nigra pars compacta (SNc), which innervates basal ganglia and leads to loss of dopamine levels in the striatum [239,240]. Although cell death mechanisms are still unknown, great attention has been focused on α-synuclein because it is the major constituent of Lewy bodies, a principal hallmark of PD. In fact, the spread of fibrillar α-synuclein pathology from the brainstem to limbic and neocortical structures seems to be the strongest neuropathological correlate of emerging dementia and cognitive impairment in the disease [241].

In this scenario, the increased level of oxidatively modified proteins in PD leads to the impairment of several cellular functions [242,243]. We have already reported the importance of PrP^C^ expression in modulating redox homeostasis [75]. In addition, some other common aspects suggest that PrP^C^ may exert neuroprotective functions in these diseases. We already know the importance of biochemical interactions between PrP^C^ and NMDAR or mGluR5 and their possible contribution in AD (see above). Along this line, dysregulation of glutamate receptors plays a role in both HD and PD (reviewed in [244,245]). The excess of glutamate is associated with NDDs, and it becomes excitotoxic by chronically activating both ionotropic and metabotropic glutamate receptors. As a result, an increase in intracellular Ca^2+^ promotes neuronal injury and cell death [246,247]. So, although changes in *PRNP* expression in early stages of PD and HD are unknown, we may speculate upon a putatively positive role of PrP^C^ in inhibiting glutamate receptors in both diseases.

So, the mechanism that promotes excitotoxicity in HD is thought to be the increased redistribution of NMDAR to the extra-synaptic compartment. Indeed, NMDARs play a key role in neuronal cell death related to HD. Moreover, it has been demonstrated that mutated HTT protein leads to sensitization of the NMDAR, resulting in an increase in extracellular Ca^2+^ invading neurons and promoting excitotoxicity [245]. Furthermore, degeneration of dopaminergic neurons in SNc induces an increase in the activity of glutamatergic neurons in the subthalamic nucleus (STN) which is believed to contribute to the motor symptoms of PD. Group I mGluRs (mGluR1 and mGluR5) are widely expressed in the basal ganglia, especially at postsynaptic sites [248]. However, mGluR5 expression is higher than mGluR1. So, its role in PD motor deficits has been shown in a variety of preclinical studies [249,250]. In fact, antagonism of this receptor ameliorates motor deficits in animal models of PD [251,252]. In this line of research, a new topic of debate is emerging: the possible intervention of PrP^C^ in inducing cognitive impairment through mGluR5 and NMDAR in synucleinopathies. As reported regarding ADDLs-PrP^C^ interaction, Outeiro’s group has indicated that PrP^C^ acts as a receptor for neurotoxic effects of oligomeric α-synuclein [23], although recent results contradict this [67].

As indicated above, neuroprotective PrP^C^ cleaved-fragment PrPN1 binds to and antagonizes the toxicity of β-sheet rich oligomers. In this line, Wetzel’s group has reported a complex aggregation pathway for a polyQ containing the N-terminal 17 amino acids of HTT exon 1. In addition, they show the intermediate structures formed during aggregation of peptides [253]. A previous study showed a protective effect of PrP^C^ in HTT pathology by reducing aggregation and associated toxicity in neuronal cells [139]. The authors suggest that PrP^C^ protects cells from a reduction in proteasome activity by maintaining levels of ROS, thereby helping to prevent protein aggregation. It would be of interest to learn about the roles of full-length PrP^C^ protein and cleaved fragments of the protein in the same model. In contrast, HD is considered a four-repeat tauopathy with tau nuclear rods [254]. In addition, GSK3β inhibitors prevent cellular toxicity caused by HD mutation [255]. Taking all the evidence together, it is tempting to posit that PrP^C^ may have alternative roles in the disease through regulation of tau levels and modulation of GSK3β activity.

Despite the lack of research about intervention of PrP^C^ in the α-synuclein aggregation process, there has been reported to be an increased tendency toward aggregation after oxidation of γ-synuclein, another member of the family that seeds α-synuclein aggregation [256]. In this context, an indirect role of PrP^C^ in this process is plausible. Also, we and others have shown that PrP^C^ is involved in the propagation and spreading of protofibrils of α-synuclein, with binding between the two proteins in *Prnp*-transfected HEK293 cells though residues located in the CC2 domain of PrP^C^ [47,157,257]. Surprisingly, these are the same amino acids (95–110) involved in binding with Aβ [213]. Since *Prnp* expression is not mandatory for α-synuclein transport in the mouse brain [258], it is tempting to consider that role in the update and transport to be a collateral effect on the principal neuroprotective role of PrP^C^ in the disease. In this respect, we also must recall that both our group and Aulic et al. [157] indicated that PrP^C^ is a receptor for the fibrillar forms of α-synuclein [21,257,259]. However, as it has been reported that PrP^C^ does not bind to oligomeric species of α-synuclein [67] in contrast to [23], additional studies are needed to ascertain whether oligomeric α-synuclein also mediates similar effects to ADDLs though PrP^C^ interaction.

**Figure 5:**
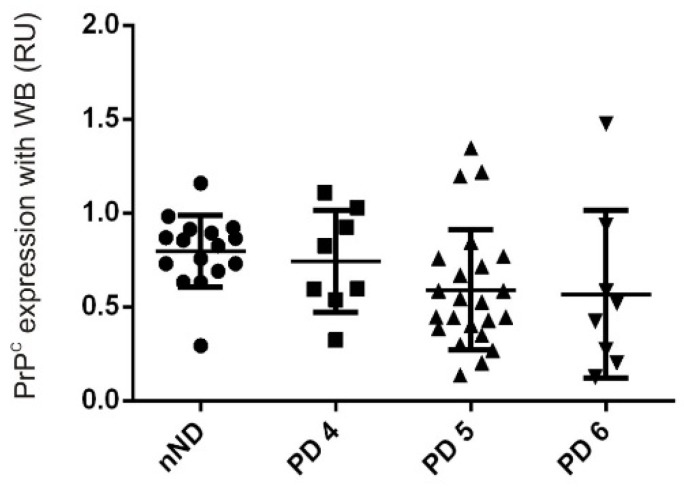
Graph representing densitometric study of PrP^C^ immunoblot analysis in postmortem frontal cortex (Brodmann area 8) from PD patients at different stages compared to non-neurodegenerative cases (nND). Postmortem brain tissue was obtained from Hospital Clinic Brain Bank, following the Code of Ethics of the World Medical Association and the protocols of the local ethical committee. Each plot represents a quantitative level of PrP^C^ standardized with actin level for each case. Data shows a progressive, albeit nonsignificant decline in PrP^C^ levels in accordance with advance of the disease. Statistical analysis of the resulting data was performed using Anova (Kruskal–Wallis with Dunn multiparametric test) and Prism 8.0 (GraphPad Software, San Diego, CA, USA).

Finally, unpublished studies by our group indicate a tendency toward decreased levels of PrP^C^ protein levels in the frontal cortex (area 8) in advanced PD patients (Braak stages 5 and 6) (Figure 5). These results may signal the importance of the protein in the progression of the pathology, as occurs in AD (see above).

## 4. Concluding Remarks

The relationship between PrP^C^ and other amyloids (oligomeric (ADDLs) and fibrillar forms (i.e., α-synuclein)) has been well established, and different roles of PrP^C^ in AD have been described (see above). We argue for the role of PrP^C^ in preventing the detrimental effects of the oligomeric species, especially at early stages of the neurodegenerative processes. In this review, we have focused our attention on analyzing a number of mechanisms through which PrP^C^ may act as a neuroprotective molecule. In fact, we must not lose sight of the progression of the protein and its derivate fragments (PrPN1, for instance) in the evolution of diseases. A correlation of the symptoms with levels of PrP^C^ expression may be an important element in increasing our understanding of the natural functions of the protein. In this review, we have also speculated about other diseases of which we do not have so much data, but regarding which it is nonetheless reasonable to posit that PrP^C^ is expressed in order to slow down disease progression. Despite this, we cannot rule out the possibility that enhanced interaction between PrP^C^ and the other proteins implicated in NDDs, such as Aβ and α-synuclein, results in fatal effects. Thus, a plausible intervention to avoid the progression of these diseases may involve blocking these specific interactions, thereby allowing the protein to maintain its natural function.

## Figures and Tables

**Table 1:** Studies on contribution of PrP^C^ to potential neuroprotection in neurodegenerative diseases.

Disease	Finding	Model	Role of PrP^C^	Key Reference(s)
**Alzheimer’s disease**	Inhibition of BACE1	In vitro	Decreases Aβ production	[158,159]
	Binding of PrPN1 to Aβ	In vitro	Blocks transformation into ADDLs	[160,161]
	Binding to STI1	In vitro	Decreases ADDLs toxicity	[162]
	Binding to Zn^2+^	In vitro	Decreases Aβ aggregation	[163]
	Binding of PrPN1 to ADDLs	In vivo	Decreases ADDLs toxicity	[164]
	Prevention of cell death by Aβ	In vivo	Decreases caspase-3 and Bax/Bcl2 levels	[165]
	Increase in PrPN1 production in brain patients	Human samples	Blocks transformation into ADDLs	[160]
	Increase in brain regions prone to oxidative stress	Human samples	SOD and GR activity regulation	[166]
	Increase in initial stages of the disease	Human samples	Downregulates tau levels	[167]
**Huntington’s disease**	Increase in proteasome activity	In vitro	Decreases HTT aggregation and toxicity	[139]
**Amyotrophic lateral sclerosis**	Induction of neuronal and glial survival signaling	In vivo	Antioxidant	[168]
**Nonspecific disorder**	Binding to Cu^2+^	In vitro	Antioxidant	[75]
	Modulation of SOD	In vitro	Antioxidant	[86]
	Modulation of GR	In vitro	Antioxidant	[83]
	Modulation of Bax function	In vitro	Antiapoptotic	[95]
	Regulation of Ca^2+^ homeostasis	In vitro	Reduces excitotoxicity	[101]
	Inhibition of NMDAR	In vitro	Reduces excitotoxicity	[108,109,169]
	PrP113-128 peptide	In vitro	Activates cAMP/PKA and MEK/Erk pathways	[116]
	PrP-Fc signaling	In vitro	Activates PI3K/Akt pathway	[75]
	Binding to STI1	In vivo	Inhibits GSK3β activity and activates 7nAChR. All together induces neuroprotective signals.	[120,122,123,170]
